# Developing tailored intervention strategies for implementation of stratified care to low back pain with physiotherapists in Nigeria: a Delphi study

**DOI:** 10.1186/s12913-023-09123-1

**Published:** 2023-02-09

**Authors:** Mishael Adje, Jost Steinhäuser, Kay Stevenson, Chidozie Mbada, Victor Alonge, Sven Karstens

**Affiliations:** 1 Therapeutic Sciences, Trier University of Applied Sciences, Trier, Germany; 2 Institute of Family Medicine, University of Luebeck, Luebeck, Germany; 3 The Impact Accelerator Unit, The Medical School Keele University, Keele, United Kingdom; 4 Department of Health Professions, Manchester Metropolitan University, Manchester, United Kingdom; 5 Department of Physiotherapy, Exercise and Sports, LUNEX International University of Health, Differdange, Luxembourg; 6 Therapeutic Sciences, Department of Computer Science, Trier University of Applied Sciences, Trier, Germany

**Keywords:** Delphi survey, Complex prognostic factors, Implementation strategies, Stratification, STarT-Back Approach, Musculoskeletal care

## Abstract

**Background:**

Stratified care approach involving use of the STarT-Back tool to optimise care for patients with low back pain is gaining widespread attention in western countries. However, adoption and implementation of this approach in low-and-middle-income countries will be restricted by context-specific factors that need to be addressed. This study aimed to develop with physiotherapists, tailored intervention strategies for the implementation of stratified care for patients with low back pain.

**Methods:**

A two-round web-based Delphi survey was conducted among purposively sampled physiotherapists with a minimum of three years of clinical experience, with post-graduation certification or specialists. Thirty statements on barriers and enablers for implementation were extracted from the qualitative phase. Statements were rated by a Delphi panel with additional open-ended feedback. After each Delphi round, participants received feedback which informed their subsequent responses. Additional qualitative feedback were analysed using qualitative content analysis. The criteria for consensus and stability were pre-determined using percentage agreement (≥ 75%), median value (≥ 4), Inter-quartile range (≤ 1), and Wilcoxon matched-pairs test respectively.

**Results:**

Participants in the first round were 139 and 125 of them completed the study, yielding a response rate of 90%. Participants were aged 35.2 (SD6.6) years, and 55 (39.6%) were female. Consensus was achieved in 25/30 statements. Wilcoxon’s test showed stability in responses after the 5 statements failed to reach consensus: ‘translate the STarT-Back Tool to *pidgin* language’ 71% (*p* = 0.76), ‘begin implementation with government hospitals’ 63% (*p* = 0.11), ‘share knowledge with traditional bone setters’ 35% (*p* = 0.67), ‘get second opinion on clinician’s advice’ 63% (*p* = 0.24) and ‘carry out online consultations’ 65% (*p* = 0.41). Four statements strengthened by additional qualitative data achieved the highest consensus: ‘patient education’ (96%), ‘quality improvement appraisals’ (96%), ‘undergraduate training on psychosocial care’ (96%) and ‘patient-clinician communication’ (95%).

**Conclusion:**

There was concordance of opinion that patients should be educated to correct misplaced expectations and proper time for communication is vital to implementation. This communication should be learned at undergraduate level, and for already qualified clinicians, quality improvement appraisals are key to sustained and effective care. These recommendations provide a framework for future research on monitored implementation of stratified care in middle-income countries.

**Supplementary Information:**

The online version contains supplementary material available at 10.1186/s12913-023-09123-1.

## Introduction

Low back pain (LBP) is highly prevalent and substantially increasing globally as the population ages. LBP cuts across national and socio-economic boundaries [1–3] and it is a leading cause of disability that has significant impact on productivity and personal life [1, 2]. Despite treatment efforts, many acute LBP cases turn chronic, and the prevalence and recurrence levels remain high, even exponentially increasing [3, 4]. Research shows that there is a presence of psychosocial risk factors in over 60% of patients with chronic non-specific LBP which further impedes outcome [5].

Research has shown that standardized risk-specific stratified treatment approaches could be superior to traditional physiotherapy approaches for patients with LBP [6–8]. These studies recognise the heterogeneous population of patients with LBP and recommend the creation of patient prognostic profiles based on potential individual responses to specific treatments [8]. Further recommendations on management strategies integrate physical and psychological treatment approaches to address psychosocial risk factors and reduce other obstacles to recovery [9, 10].

Stratified care (SC), is an approach that involves differentiating and targeting prognostic subgroups, aligning the risk of an unfavourable treatment outcome with specific evidence-based treatment procedures [11, 12]. It is described as best practice in multiple international guidelines [13, 14]. A comprehensively evaluated procedure is the Subgroups for Targeted Treatment (STarT-Back) approach. It has shown the potential to improve treatment outcomes, patient and clinician satisfaction and reduce the cost burden [15–17].

The STarT-Back tool (SBT) is one of several tools developed, translated and cross-culturally adapted to assist this prognostic profiling [18, 19]. The method of stratification using SBT is unique because it matches specific treatments to subgroups of patients with similar characteristics i.e. complex prognostic factors, categorising patients into low, medium or high risk subgroups [15]. Patients with LBP in the low-risk subgroup receive care involving reassurance, medication and self-management advice to discourage other speciality treatments including x-ray requests; patients in the medium-risk subgroup, receive support using evidence-based conservative treatments offered by physiotherapists, further preventive measures against future LBP related disability and for patients in the high-risk subgroup, psychologically informed physiotherapy treatment (PIP) [20]. The STarT-Back (SB) approach borrows these components of self-management and patient-centred approach principles in a bid to provide more tailored treatment and optimize LBP outcomes [20].

Identifying prognostic subgroups of patients and allocating specific treatment content as recommended is currently not utilized in low/medium resource countries like Nigeria when planning treatment [21]. There are well-known and available evidence-based treatment methods such as exercises, manual therapy and analgesic medications [22–26]. In spite of this, the general physiotherapy approach to the treatment of LBP in low-medium income countries involves a variety of largely non-evidence based modalities. One study reported heat therapy was most commonly used for 1 in every 2 cases, followed by exercise therapy and then education/advice which was not only significantly low in frequency but included education on the diagnosis of LBP and reducing activities for sufferers [27]. These methods are used in combination or separately to treat patients with LBP [28]. Treatment modality, intensity and duration are not influenced by prognostic factors and strict use of guidelines is not popular [29]. For a large percentage of the rural population, there is also the practice of traditional bone setting; a ‘method of traditional medicine’ learned by apprenticeship and using herbs and roots for treatment and pain relief. The reported high patronage (> 70%) is due to the immense faith placed on them by the people, its relatively low cost and easy access [30].

This might be in contrast to high-income countries where more advanced care options with broader applications are available as mainstream or adjunct care options for LBP and its complications or related comorbidities [31–33]. Since LBP is a complex multi-faceted phenomenon, it benefits from multimodal interventions some of which might be unavailable in low/medium resource countries [25].

Recent studies reveal that using the SC model enhances practice and garnered a positive perception among clinicians, patients and general practitioners. It also reveals contextual concerns that might serve as barriers to implementation such as time constraints, treatment expectations held by patients regarding the method and success of approach and the incentivised tradition of practice. These studies reveal potential facilitators like communication, patients’ trust and socio-cultural factors with the potential to accentuate implementation [17, 34].

There still exists the need for developing consensus on strategies that can be adopted for a tailored implementation of SC for LBP among physiotherapists in low-medium income countries. Studies have recommended that for tailoring interventions, a stepwise process should be adopted [35, 36]. This involves first identifying the determinants affecting implementation, then developing strategies for utilising facilitators and overcoming barriers by consensus [35]. This study aims to identify tailored strategies, approaches and adaptations to address barriers and promote enablers to the implementation of SC with Nigerian physiotherapists.

## Materials and methods

The Web-based Delphi technique chosen for this study is a structured iterative process that provides an accurate synthesis of opinions gathered from panellists through multiple rounds aiming for group consensus [37, 38]. The key components are anonymity and iterative feedback to participants [39]. This procedure was chosen because of the comparative advantage it offers in gathering subjective information from a group of panellists on a particular subject and is especially reliable when they are not physically present [40]. This Delphi study took place from November 2021 to July 2022.

### Participants

The participants were a panel of physiotherapists sampled following the criteria for panellists described by Hora [41] and Trevelyan [42]. Based on this, the inclusion criteria allowed physiotherapists with a minimum of 3 years of clinical experience, with postgraduate degrees or Continuous Professional Development courses (CPDs) and available and willing to participate in the study throughout its entire duration. All participants were physiotherapists licenced, registered above 18 years and practicing in Nigeria. Physiotherapists who did not meet any of the panellist requirements in this study were excluded. An expected sample size of 60 participants was planned for this study providing sufficiently representative data for the targeted heterogeneous population [37], fitting to the aim of the study and allowing for attrition as exemplified by similar research studies [43, 44].

Potential participants who met the baseline inclusion criteria were contacted via email. These physiotherapists were registered with the regulatory board of physiotherapists, had completed a recognised training programme, and working in Nigeria. Potential participants received a recruitment link, explaining the Delphi process and its objectives [37]. They were also informed about the study, anonymity and requested to confirm their qualification on each of the inclusion criteria before consenting to the study. An incentive of 2 Euro worth of phone credit vouchers was provided to reduce the attrition rate as recommended in the literature [45].

### Data collection

#### Procedure

Potential participants were invited to a nationwide interactive physiotherapy webinar on stratified care to stimulate their interest. They were further informed about stratified care via tailored interactive videos before participation. This included the rationale for SB approach, psychosocial barriers to recovery, using and scoring the SB Tool and matched treatments, scientific underpinning of the approach, clinical and economic benefits of the SB approach obtained from literature [11, 12, 15].

**Fig. 1:**
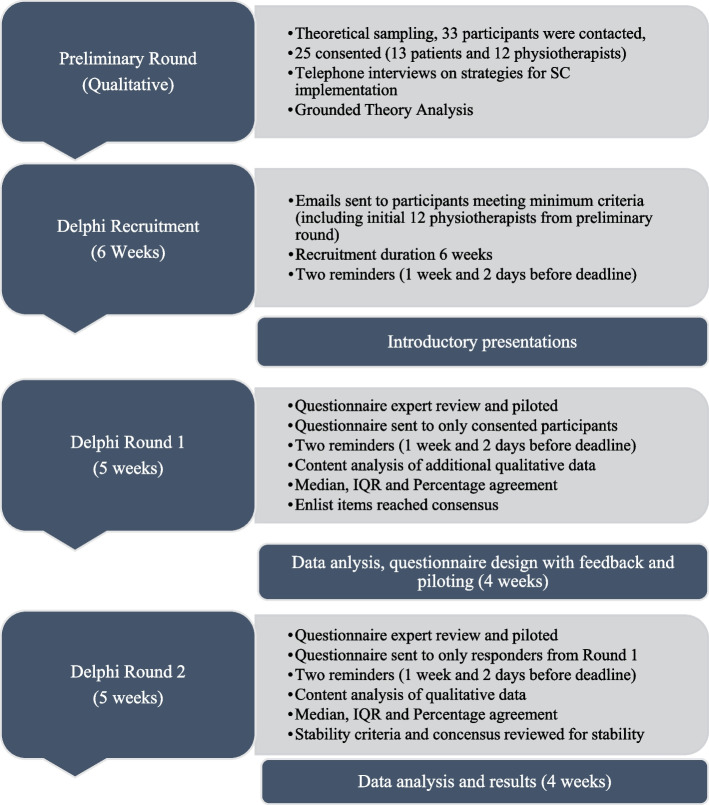
Data collection procedure. Legend: SC: Stratified Care; IQR: Inter-quartile range

A preliminary round (see Fig. 1) was carried out as recommended in the literature as a feature of Delphi studies when sufficient content is not available from previous studies as it improves responses and reduces dropout [38, 46, 47]. It was a semi-structured telephone interview, exploring barriers and enablers to implementation of stratified care. Interviews were audio recorded, transcribed and analysed using Grounded theory [48] generating categories and sub-categories used in the Delphi rounds (Additional file 1). This preliminary round was described in detail in a published manuscript [34].

*Round 1:*After a recruitment phase, round 1 was carried out. The aim of this round was to investigate the level of agreement on statements derived from the qualitative preliminary round. Participants who responded to the recruitment email were contacted for this round. The survey was presented to participants via an online link for rating according to their level of agreement. Qualitative feedback options were provided for participants to give additional strategies.

*Round 2:*After analysis of round 1 data, round 2 questionnaire was developed and distributed. This round aimed to investigate the level of agreement between the participants regarding the aspects of the previous phase. This was sent only to participants who responded to the first round [49]. Participants were urged to reconsider their original responses and rate the statements in the light of the feedback from the first round and grade again. At each stage, participants were free to change their opinions if they wish and were given the possibility to explain the reasons if any. The criteria for Stability and consensus were considered.

In both rounds, non-responders were reminded via email one week and 2 days before the deadline. Participants were blinded to each other but known to the researcher, however, contact was provided for private feedback and participants were given 5 weeks to complete this round. There was a period of 4 weeks interval between rounds.

### Questionnaire design

The questionnaire was derived from perceived barriers and enablers to implementing stratified care identified in the preliminary round with additional opinions provided by participants (Fig. 1). Terminologies from the preliminary round were maintained as closely as possible to reflect the original content suggested by participants [37]. To fulfil the criteria for an acceptable validation process in a Delphi study [50], statements selected to make up the questionnaire were structured following the recommendations for word count and complexity [51].

These statements were further prepared for grading on a 5-point Likert scale (from ‘strongly disagree’ to ‘strongly agree’) with a neutral midpoint option, ascending response options, fully labelled response options, response options in a horizontal format provided to participants as recommended and merged with open-ended questions allowing the participants freedom to bring fresh views [45].

To ease comprehension, statements with similar ideas making up the questionnaire were classed together forming three categories; i) strategies to best modify stratified care management to fit the National context, ii) views on how training and education can best be done to help the implementation of stratified care, iii) ideas on conditions necessary to enhance the implementation of stratified care nationally. The questionnaire was then reviewed for language, content and sequencing by the research team consisting of physiotherapists with experience in SC and Delphi methodology.

The final resulting questionnaire made up of 30 statements hosted on an online survey platform [52] was pilot-tested with five individuals using the Think-aloud method [53]. During this piloting process, the participants were asked to have feasibility and importance in mind while responding to the questions. Hence the questionnaire was further modified to contain these components before presentation to the panellists in Round 1 of the Delphi process.

For the second Delphi round, the questionnaire from round 1 was further modified based on the previous responses. This contained all thirty questions in the same classification as the previous phase and a statistical summary of answers with the possibility to provide further comments. This was based on recommendations from literature to ‘help motivate panel’ as they see that the process actually worked also giving participants the opportunity to reflect on their previous judgement [37, 42].

### Data analysis

*Rounds 1 and 2*: Descriptive statistics were employed to present participants' sociodemographic data. For participants responses to statements, pre-set measures of central tendency (median) and measures of dispersion (Interquartile range-IQR) were employed. To calculate the median and IQR, the response options were assigned numerical values. For the options 1 to 6 in ascending order, the numerical values were; ‘strongly disagree’ (1), ‘disagree’ (2), ‘neutral’ (3), ‘agree’ (4), ‘strongly agree’ (5), ‘no opinion’ (0). Wilcoxon matched-pairs signed rank test was used to determine the degree of stability for each round [54]. Statistical results of each phase were fed back to participants. Qualitative data from the additional opinions were analysed using qualitative content analysis [55].

Inter-round stability and consensus were considered suitable termination criteria as recommended in literature [56]. Inter-round stability was considered based on recommendations by Dajani et al. [57]. Inter-round stability of statements reveals the degree to which participants' responses are changing or not changing. Statements were considered stable if the median and interquartile range of responses from all participants did not change significantly between rounds [57, 58]. Wilcoxon matched-pairs signed-ranks test was used to assess the stability of the responses between stages as used in a previous Delphi study [54].

The consensus was determined using IQR (≤ 1) and median (≥ 4) [59]. In addition, percentage agreement was described as the percentage of panellists who respond “agree or strongly agree” to an individual statement. Each statement that receives ≥ 75% agreement was considered as having reached a consensus [60]. Statements were further described by level of agreement, this means that consensus statements in the top 5% with the highest percentage of agreement was considered the most important [29]. Following these pre-defined criteria, two rounds were sufficient as exemplified in a study [49]. The study report was prepared following the recent guidelines for reporting Delphi techniques in Health Science research [61].

## Results

**Fig. 2:**
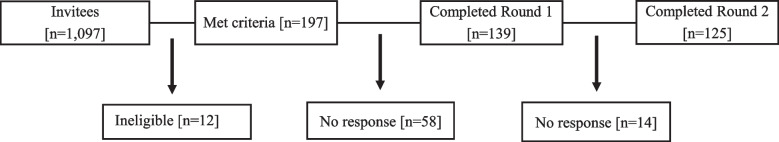
Study flowchart

A total of 1,097 emails were sent to participants meeting the minimum criteria. Feedback was received from 209 participants. Due to relocation out of the country or stopping practice 12 participants were ineligible. Therapists meeting the inclusion criteria were 197. Of those, 139 completed the first round and 125 (90%) completed the second round after receiving the link to the questionnaire as seen in Fig. 2.

Table 1 below shows further details on participants demographics. Their mean age was 35.2 (SD 6.6), 39.6% were female and 1 participant was diverse. The majority 47 (33.8%) had above 5 and below 10 years of clinical experience, 76 (54.7%) had BSc as their highest educational level, and 80 (57.6%) had musculoskeletal area of interest. Participants who worked in the teaching hospital and federal medical centres made up 54 (38.8%).

**Table 1:** Characteristics of the study population

Characteristics (*n* = 139)	n (%)	Mean (SD)
**Sex**		
Male	83 (59.7)	
Female	55 (39.6)	
Diverse	1 (0.7)	
Age (in years)		35.2(6.6)
**Years of experience with low back pain**		
Up to 5 years	32(23.1)	
> 5 years to 10 years	47(33.8)	
> 10 years to 15 years	38(27.3)	
> 15 years to 20 years	16(11.5)	
> 20 years to 25 years	4(2.9)	
> 25 years to 30 years	1(0.7)	
> 30 years to 35 years	1(0.7)	
**Qualification**		
BSc/BMR(PT)	76(54.7)	
M.Sc	47(33.8)	
Ph.D	11(7.9)	
Other	2(1.5)	
DPT	1(0.7)	
Diploma	2(1.4)	
**Areas of Interest**		
Cardiopulmonary	19(13.7)	
Community physiotherapy	32(23.0)	
Ergonomics and Occupational	21(15.1)	
Geriatrics	21(14.4)	
Neurology	42(30.2)	
Oncology/Palliative care	10(7.2)	
Musculoskeletal	80(57.6)	
Paediatrics	24(17.3)	
Sports	36(25.9)	
Women's health	25(18.0)	
**Work setting**		
Primary health care	5(3.6)	
Teaching hospital and federal medical centres	54(38.8)	
General/state hospital	21(15.1)	
Specialist hospital	19(13.7)	
Home and community physiotherapy	26(18.7)	
Physiotherapy training institute (university)	15(10.8)	
Sports centre	8(5.8)	

Legend: *BSc* Bachelor of Science, *MSc* Master of Science, *PhD* Doctor of Philosophy, *PT* Physiotherapist

*Round 1*: In this round, participants completed the rating of 30 statements and responded to the open-ended questions put forward. Of these 30 statements, 25 reached the criteria for consensus as seen in Table 2. There were 5 statements that failed to reach consensus; statement 5 (72%), statement 9 (61%), statement 15 (35%), statement 26 (73%) and statement 28 (70%) with the IQR ≥ 2 for each of the 5 statements.

**Table 2:** Results from Round 1 and Round 2 Delphi processes

**Statements:** To implement Stratified Care,	**Rounds**	^**a**^**C1**	^**b**^** C2**		^**c**^**Stability (Wilcoxon)**
		^**d**^**Agreement (%)**	**Median**	**IQR**	
1. Start with senior PTs and let them supervise their junior colleagues	Round 1	88	4	1	
2. Hold quality improvement meetings to review successes and adherence	Round 1	96	5	1	
3. Educate the patients while treating them to save time	Round 1	94	5	1	
4. Schedule assessment and treatment on two separate days	Round 1	76	4	1	
**5. Translate the SBT into Nigerian pidgin.**^**e**^	Round 1	72	4	2	0.767
	Round 2	71	4	2	
6. Government should set up an affordable health insurance system	Round 1	90	5	1	
7. Increase salary increments for PTs who train and practice PIP	Round 1	84	4	1	
8. Seek co-operation of the hospital administration	Round 1	79	4	1	
**9. Implementation efforts should begin with government hospitals. **^**e**^	Round 1	61	4	2	0.109
	Round 2	63.7	4	2	
10. Patients should visit clinics with time and training on PIP	Round 1	87	4	1	
11. Include training on PIP for undergraduate PTs	Round 1	96	5	1	
12. Train PTs on pain-relieving medications	Round 1	77	4	1	
13. Adopt standardised LBP treatment guideline in Nigeria	Round 1	93	5	1	
14. Group monitoring of colleagues is needed	Round 1	82	4	1	
**15. TBS should share knowledge and ideas with PTs. **^**e**^	Round 1	35	3	3	0.676
	Round 2	35.4	2	3	
16. Training workshops on PIP for licenced physiotherapists	Round 1	94	5	1	
17. PT speciality groups should take the responsibility of advocacy	Round 1	85	4	1	
18. Inform other health professionals about SC	Round 1	91	5	1	
19. Modify patients’ expectations by educating them on expected outcomes	Round 1	96	5	1	
20. Use public media sources to dispel false information on LBP	Round 1	93	5	1	
21. Encourage patients to learn about their own condition	Round 1	91	5	1	
22. Monitor patients’ self-care routine	Round 1	91	5	1	
23. PTs self-examination to remove wrong LBP beliefs and attitudes	Round 1	90	5	1	
24. Use research results to convince PTs colleagues on SC	Round 1	93	5	1	
25. Allocate sufficient time for PT-patient communication	Round 1	95	5	1	
**26. Patients should get a second opinion on the clinician’s advice. **^**e**^	Round 1	73	4	2	0.239
	Round 2	63	4	2	
27. Print STarT-Back Questionnaires for routine use in waiting rooms	Round 1	92	5	1	
**28. Physiotherapy consultations should be carried out online. **^**e**^	Round 1	70	4	2	0.406
	Round 2	65.3	4	2	
29. Provide an electronic version of the STB and fill online	Round 1	89	4	1	
30. Patients should fill the SBT at home	Round 1	80	4	1	

Legend: *TBS*: Traditional bone setters, *PIP* Psychologically Informed Physiotherapy, *LBP* Low back Pain, *SC* Stratified care, *SBT* STarT-Back Tool, *PT* Physiotherapist

^a^ C1 (Consensus criteria 1): ≥ 75% agreement

^b^ C2 (Consensus criteria 2): Median ≥ 4 and Inter-Quartile-Range (IQR) ≤ 1

^c^ Stability criteria: Wilcoxon matched-pairs signed-rank test was calculated for statements which did not reach consensus in the first round

^d^ Agreement: Selection of response options *4—agree* or *5—strongly agree*

^e^ Statements not reaching consensus in Round 1, progressed to Round 2 and tested for stability

^*^Significance level is set at p ≤ 0.05

*Round 2*: In this round, the same 5 statements as in round 1 failed to reach consensus; statement 5 (71%), statement 9 (63.7%), statement 15 (35.4%), statement 26 (63%) and statement 28(65.3%). The IQR remained at ≥ 2 for each of the 5 statements. The Wilcoxon’s matched-pairs signed-rank test shows no significant difference in the 5 statements between round 1 and round 2 with alpha level set at *p* < 0.05. Table 2 displays all 30 statements including the results from Wilcoxon’s matched-pairs signed-rank test, percentage agreement, median and inter-quartile range. Further details on individual item responses are shown in Additional file 2.

When arranged in order of importance, the top 5% of the items reaching consensus ranked by percentage of agreement were 4 statements, these statements had the highest levels of agreement (> 95%). These statements were; Modify patients’ expectations by education (96% agreement), Holding quality improvement meetings to review successes and adherence of the approach (96% agreement), Psychosocial care training for physiotherapists at undergraduate levels (96% agreement) and allocating time for patient communication (95% agreement) as shown on Table 3.

**Table 3:** Statements that attained consensus ranked in the level of importance

Rank	Statement	Consensus Criteria 1	Consensus Criteria 2	
		**Agreement (%)**	**Median**	**IQR**
**1)**	**Modify patients’ expectations by educating them on expected outcomes.**^**a**^	**96**	**5**	**1**
**2)**	**Hold quality improvement meetings to review successes and adherence.**^**a**^	**96**	**5**	**1**
**3)**	**Include training on PIP for undergraduate PTs.**^**a**^	**96**	**5**	**1**
**4)**	**Allocate sufficient time for PT-patient communication.**^**a**^	**95**	**5**	**1**
5)	Training workshops on PIP for licenced physiotherapists	94	5	1
6)	Educate the patients while treating them to save time	94	5	1
7)	Adopt standardised LBP treatment guideline in Nigeria	93	5	1
8)	Use public media sources to dispel false information on LBP	93	5	1
9)	Use research results to convince PTs colleagues on SC	93	5	1
10)	Print STarT-Back Questionnaires for routine use in waiting rooms	92	5	1
11)	Encourage patients to learn about their own condition	91	5	1
12)	Monitor patients’ self-care routine	91	5	1
13)	Inform other health professionals about SC	91	5	1
14)	Government should set up an affordable health insurance system	90	5	1
15)	PTs self-examination to remove wrong LBP beliefs and attitudes	90	5	1
16)	Provide an electronic version of the SBT and fill online	89	4	1
17)	Start with senior PTs and let them supervise their junior colleagues	88	4	1
18)	Patients should visit clinics with time and training on PIP	87	4	1
19)	PT speciality groups should take the responsibility of advocacy	85	4	1
20)	Increase salary increments for PTs who train and practice PIP	84	4	1
21)	Group monitoring of colleagues is needed	82	4	1
22)	Patients should fill the SBT at home	80	4	1
23)	Seek co-operation of the hospital administration	79	4	1
24)	Train PTs on pain-relieving medications	77	4	1
25)	Schedule assessment and treatment on two separate days	76	4	1

Legend: *PIP* Psychologically Informed Physiotherapy, *LBP* Low Back Pain, *SC* Stratified Care, *SBT* STarT-Back Tool, *PT* Physiotherapist

^a^ Statements reaching consensus and selected as the most important (top 5%) for implementation of Stratified Care

### Qualitative responses

The responses and additional suggestions from rounds 1 and 2 fit well into the scope of categories previously derived (14 already identified categories in the preliminary round giving rise to the initial questionnaire), hence it was not necessary to add any new suggestions/questions to the questionnaire. They were re-classified under the 3 major existing categories of the questionnaire with accompanying definitions arising from participants’ responses (Additional file 3); Strategies to best modify stratified care management to fit the national context, Strategies on how training and education can best be done to help the implementation of stratified care, and Strategies related to conditions necessary to sustain the implementation (Table 4).

**Table 4:** Qualitative data for Rounds 1 and 2: Categories and Sub-categories

Category	Sub-category
A) Strategies to best modify stratified care management to fit the national context	Hierarchal implementation
	Approach modification
	Tool modification
B) Strategies on how training and education can best be done to help the implementation of stratified care in Nigeria	PT training
	Educating patients
	Patient Communication
	PT practice standardisation/regulation
	Re-adjusting patients’ expectations
C) Strategies related to conditions necessary to sustain the implementation	PT attitude re-adjustment
	Treatment tradition
	Process modification
	Management solutions
	Interprofessional collaborations
	Financial solutions

Legend: *PT* Physiotherapist

#### Strategies to best modify stratified care management to fit the National context

This category describes the unique challenges facing the national Health Care context, advantages which can be harnessed for the implementation of SC and peculiar ways of modifying the approach and tool to enhance suitability.

Here some participants commented on several issues they felt could help with contextual adaptation. A vital aspect was the suggestion on pilot *implementation through the senior colleagues*, Heads of departments and *intermittent meetings/quality improvement monitoring* how the approach progresses. This form of *hierarchal implementation* was seen by some responders as a viable means for implementation. In addition, some suggested ways to optimise the tool and approach by automation, *using electronic versions of the tool* and *creating more translations*.

‘High level PT mentorship needed’ *(F/ < 5/MSc/TeachHosp). [*Format: Quote (Gender/Qualification/Years of experience/Work)]

‘Periodic review of the approach should be done per time and encourage PTs in using the strategy’ (M/BSc/5–10/GenHosp).

‘Other language version of the SB tool should be made available for easy application because of the illiteracy level of many patients around the country’ (F/Msc/15–20/TeachHosp).

#### Training and education to aid the implementation of stratified care

This category relates to how education and training can be best carried out for stakeholders to aid the implementation of SC in clinical practice.

Some participants felt that *undergraduate training* should be carried out in areas related to *patient communication* and psychologically informed physiotherapy. These ones felt such training should be compulsory for every physiotherapist in training as this was very important in *equipping them handle patients’ expectations*. It helped with their confidence, *standard of practice* and enhances their knowledge. For patients, others felt rural outreaches can be easily done where communities are given educational seminars which should contain pain beliefs.

‘There should be compulsory training for PTs on stratified care approach’ (F/MSc/5–10/SpecialHosp)

‘PT undergraduates should be allowed to experience SC during their internship when practiced by licensed PTs’ (F/BSc/5 > /TeachHosp).

‘Reaching to rural communities to deliver health talks’ (M/MSc/10–15/TeachHosp).

#### Strategies related to conditions necessary to sustain the implementation

This category deals with the criteria that therapists feel are necessary to be in place for a smooth and sustained delivery and utilisation of SC. These conditions need to be ongoing concurrently with implementation and should serve to prepare stakeholders to accept this approach.

In order to ensure smooth implementation, some physiotherapists reported that *interprofessional collaborations* have to be made. Also, hospital managements have to be involved, to ensure sufficient PTs are employed, and remunerated and ultimately reduce workload. This can ensure sufficient *time for patient communication* relevant for all prognostic risk groups in the STarT Back classification system. The *treatment tradition* and *attitude of PTs* will change gradually with good results stemming from the pilot use of SC in practice.

‘Traditional ways will improve and be refined gradually with use’ (F/BSc/5–10/SportCentr).

‘More PTs should be employed in hospitals to reduce the burden of work and thus enable PTs to have more time for assessment and treatment’ (M/PhD/10–15/TrainInst).

‘Careful involvement of other health personnel participating in the client care’ (F/Bsc/ < 5/PriHC).

## Discussion

This study developed strategies for the implementation of stratified care in Nigeria after two rounds of Delphi survey. The aspects of undergraduate training, sufficient time for patient-clinician communication, modifying patients’ expectations by educating them on expected outcomes and quality improvement meetings received consensus by physiotherapists as the most important and feasible strategies.

In this study, participants rated the statements based on their level of importance and feasibility within the context of all statements [37]. Participants highlighted broad strategies for implementation of a stratified model of care for patients with low back pain. These areas include; Educating and sharing information with stakeholders, inter-disciplinary collaboration and good patient communication. Undergraduate competency training aimed to adjust physiotherapists' beliefs and hone their skills in managing psychosocial risk factors in patients with complex conditions. Internal appraisals of the approach should be carried out to ensure compliance. Time scheduling was highlighted and categorised to help participants and was seen to have achieved the highest consensus. These aspects were consistent with the literature [17]. However, findings from this study reveal that of these areas, four statements were considered contextually most critical and feasible for implementation.

### Overcoming patient expectations using education

Delphi panellists agreed that implementation needed physiotherapists to modify patients’ expectations by educating them on expected outcomes. Evidence from research shows that patients’ expectations when drawn from an inaccurate understanding of pain, could impede outcome if used as indices to measure satisfaction and a guide to treatment choices [62, 63]. For patients classified by the SB approach into low risk and high risk sub-categories, this strategy is of particular importance due to the recommended one-off treatment session, extended consultation and advice components. An understanding of the rationale and quality of the SB intervention will help patients trust the outcomes (e.g. that the one-off session for low risk sub-group is sufficient or that staying active in spite of pain is the right approach), establish reasonable expectations and re-focuses the patients’ attention on the treatment goals [64, 65]. For the delivery of education, participants in this study suggested that organising community seminars/outreaches and the use of social media platforms are effective as they reach individuals before they become patients and translate into amenable and co-operative patients [66, 67].

### Quality improvement meetings

Delphi participants affirmed that physiotherapists should hold quality improvement meetings among themselves to review successes and adherence. This is in tandem with the Consolidated Framework for Implementation Research (CFIR) framework which expounds this further by asserting that the process of implementation should be supported by engaging users, opinion leaders through a process of self-reflection, evaluation and re-evaluating of the success and areas to improve. Such appraisals are not only key to implementation, but they also ensure the sustainability of the intervention [65]. A recent study shows that implementation using quality improvement strategies have better outcomes when established as routines in clinical practice, and executed with peer assessment workshops and support systems [68]. In the global implementation of stratified care, a research work by Sowden et al. suggests that stakeholder engagement can be done through various mechanisms including quality improvement meetings and mentorship [67]. This has a bearing not only on hierarchal implementation, where the seniors physiotherapists instigate these appraisals and systematically monitor the progress, it also involves their full participation and cooperation in convincing junior physiotherapy practitioners [34].

### Undergraduate training

The Delphi survey received reasonable input from early graduates up to 5 years of clinical practice making up nearly one-fifth of the study population. Their opinions contributed to the strong consensus regarding the need for universities to include training on psychosocial care for physiotherapists at the undergraduate level. Studies testing the skills and capacity of fresh graduates in communications strategies and other aspects of cognitive behavioural treatment shows deficiencies with potential to be translated into practice. Revealing that training on psychosocial aspects including communication needs improvement [69, 70]. This important aspect of physiotherapy education is grossly limited within physiotherapy curriculum, due to limited patient contact and experiential teaching in training [70].

Therefore, undergraduate and entry-level physiotherapists should be targeted with sufficient practical components. This should be introduced early into their curriculum with adequate patient contact and close mentorship to ensure effective and sustained use of stratified care in practice and in this way to facilitate implementation [65, 71].

### Sufficient time for patient-clinician communication

Communication in this context refers to ongoing individualized dialogue to improve patient condition and foster therapeutic alliance [72]. These conversations should be aimed at learning patients’ needs, perspectives and experiences, convincing the patients on treatment options, discussing treatment goals, building rapport, trust, self-efficacy, motivation, validating patients' feelings and normalise their experiences [65, 67]. The panellists in this study agreed that such open communication between physiotherapists and patients should be given priority and more time. It was also interesting to see that strategies to save time and incur cost savings like ‘educate the patients while treating them to save time’ were not a top priority to panellists as seen in other settings. This might be due to the structured statutory cadre-based system of payment used nationwide since physiotherapists are not paid based on time spent treating patients or based on the number of patients seen, these issues could have been of little relevance giving the possibility of extended time-based care [17]. This is directly in line with the SB approach which allocates an extended 30 min consultation session for patients in low and medium-risk categories and 45 min for patients in high-risk categories. Damschroder et al. [58] further suggested that for implementation, a common ground could be used to initiate some form of ‘marketing’ to attract and involve appropriate individuals and overcome indifference or resistance. Since physiotherapists easily see the importance of quality time for communication and a reduced income from one-off consultations might be less of a challenge, this can be a starting point and highlight for introducing SB approach for implementation during nationwide advocacy programs.

Notable other statements reaching consensus were ideas cutting across broad areas essentially aimed to standardise practice, inter-disciplinary collaboration and introduction of systemic managerial changes such as revamping the health insurance and referral systems. These aspects are vital but were not among the top consensus items. One possibility could be explained by their feasibility. For instance, creating a standard framework for uniform understanding, classification and measuring of a patient's conditions was the idea behind the International Classification of Functioning, Disability and Health (ICF). Its core set for LBP represents the typical spectrum of problems in functioning for patients with LBP [73]. In a study by Kirschneck et al., physiotherapists from 32 countries largely supported the core set for low back pain relating to activities and participation, similar in concept to the SB approach [73]. However, studies show that though clinicians support it, some still find issues with feasibility and challenges in clinical practice [74].

Research shows that the healthcare systems in low-medium income countries possess unique complexities and the possibilities are few due to poor funding and dire workforce shortages. There is also the issue of health care options for patients as inequalities exist between traditional, public and private Sects. [74]. These findings reveal that physiotherapists are aware of these issues and strategies using these statements would potentially receive good support.

## Strengths and limitations

In the initial sampling, there was a response from one-fifth of the total number of contacted individuals. This implies that caution be taken in generalising the results of this study since the response rates might not represent the opinions of the entirety of the population. Further, there are some possible reasons to explain this level of response. Recent nationwide studies show a similar response ratio among healthcare workers especially when further expert criteria are required [75–77]. This may be due to the level of expertise, time and resource demands of online Delphi surveys [78–80]. Nonetheless, the recommended use of incentives, reasonable deadlines and personalised reminders in this study ensured high consistent response rates through rounds [78, 79].

The obtained demographics in this study were representative of the national physiotherapy workforce as the data shows that four-fifths of participants were less than 40 years, the majority were male with Bachelor’s degree, and working in tertiary health care institutions. This distribution can be corroborated by data from the World Physiotherapy database and demographics from recent studies [81–83].

This Delphi study was based on thorough qualitative data collected rigorously and analysed. One recent review and one methodological study concluded that concise statements lead to better quality responses and results since more than half of the Delphi studies reviewed had a maximum of 50 statements [37, 79]. There is evidence that a thorough first round based on systematically data extraction and analysis could produce shorter survey rounds [49, 79]. This was further reiterated by the qualitative data obtained from the rounds. Though the majority consisted of closed questions, the responses and suggestions after analysis resulted in sub-categories and quotes that fit into and strengthened pre-derived categories.

In this study, the questionnaire did not specify participants to respond either based on the feasibility or importance of statements. While this might be seen as a limitation, during the piloting process, the questions were cleverly modified to inherently imply feasibility. This was again checked from the pre-tests how they understood it so that while participants rated the level of importance they equally had feasibility in mind. This was piloted severally and reviewed to ensure the desired optimal outcome.

## Conclusion

The Delphi method successfully elicited key recommendations on strategies with good potential to achieve sustained implementation of the stratified model of care. Educating patients with co-developed resources aimed at modifying expectations and focusing on patient communication to normalise their experiences and facilitate self-efficacy achieved consensus as the most feasible and important strategies. Additionally, undergraduate training on communication and psychosocial factors inherent in musculoskeletal conditions provide a framework for managing these subgroups. Quality improvement meetings for clinicians can ensure sustainability of stratified care in clinical practice. How the impact of these approaches incorporated into routine practices can be objectively quantified and reflected in patient well-being might be interesting for future research.

## Supplementary Information

**Additional file 1. **Qualitative Data from Preliminary PhaseInforming Round 1 Questionnaire Development.

**Additional file 2. **Individual Item Responses in Round 1 and Round 2.

**Additional file 3. **Qualitative data for Rounds 1 and 2. Common themes and indicative quotes.

## Data Availability

All data generated or analysed during this study are included in this published article [and its supplementary information files].

## Abbreviations

STarT: Subgrouping for Targeted Treatment
SBT: STarT Back Tool
MSK: Musculoskeletal
LBP: Low back pain
IQR: Interquartile range
PIP: Psychologically Informed Physiotherapy
SC: Stratified Care
PT: Physiotherapist
TBS: Traditional bone setters
