# Supplementary material for: Developing tailored intervention strategies for implementation of stratified care to low back pain with physiotherapists in Nigeria: a Delphi study
Source: BMC Health Serv Res. 2023 Feb 9;23:134. doi: 10.1186/s12913-023-09123-1 (PMC9909884; doi:10.1186/s12913-023-09123-1)
Supplement: Supplementary file 1 — Additional file 1. Qualitative Data from Preliminary PhaseInforming Round 1 Questionnaire Development. [file 12913_2023_9123_MOESM1_ESM.docx]

**Additional file 1: Qualitative Data from Preliminary Phase Informing Round 1 Questionnaire Development.**

| **Categories** | **Sub-categories** | **Codes** |
| --- | --- | --- |
| Outlook | 1. Impression on approach | 1. SC approach would be difficult to apply in Nigeria 2. SC is applicable in government hospitals 3. SC is not applicable in private hospitals setting 4. SC will not work well in government hospital 5. Treatment timing in new approach might be a problem |
|  | 1. Impression on tool | 1. The questions are very useful 2. Questions in the SB tool are straightforward |
|  | 1. Acceptability | 1. Patients would like the idea 2. SC treatment is a welcome development |
|  | 1. Applicability | 1. SC is a feasible approach 2. SC does not cover many areas |
| Barriers | 1. Financial challenges | 1. High cost of treatment 2. Faulty health insurance system 3. Physiotherapists go for SC training at their own cost |
|  | 1. Patient attitude | 1. Patients absconding 2. Patients enjoy passive treatments 3. Patients initial resistance |
|  | 1. Interprofessional challenges | 1. Most patients are not referred early for physiotherapy 2. Other clinicians thinking physiotherapist not on the right track 3. Sensitizing other health professionals |
|  | 1. Organisational roadblocks | 1. Patient load is overwhelming 2. uncooperative management 3. Hospitals lack a blend of old and new physiotherapists 4. Unavailability of technology |
|  | 1. Physiotherapist beliefs | 1. Physiotherapists believe that more treatments work best 2. Routine is faster than using a tool 3. Physios belief that patients should reduce activity due to pain |
|  | 1. Patient belief system | 1. Clinicians are the patients last hope 2. Clinicians did the best they could 3. Clinicians learn by practice |
|  | 1. Patient expectation | 1. Patients expect pain should leave completely 2. Patients expecting more 3. Patients only feel you have done something when you touch them |
|  | 1. Socio-cultural factors | 1. Language dialects as a barrier to communication 2. Preference for trado-medical care |
|  | 1. Physiotherapist attitudes | 1. Physiotherapists have a system they know and already use 2. Physiotherapists unwilling to shift paradigm 3. Convincing physiotherapists to use approach 4. Self-reliance makes physios victims of un-wholistic practice |
|  | 1. Patient concern | 1. Patients debilitating pain 2. Repeated treatments from the hospital no sufficient result 3. Patients are unhappy with current treatment 4. Patients fears and desperation |
|  | 1. Treatment tradition | 1. Physiotherapists want to treat every patient for the financial benefit 2. Previous treatment based on diagnosis 3. Previous treatment using Electrotherapy 4. Previous treatment with massage 5. Previous treatments using Radiological examination |
|  | 1. Patients knowledge | 1. Patients are not aware of physiotherapy 2. Patients do not understand back pain 3. Patients want you to do what they already know |
| Strategies | 1. Physiotherapy practice standardisation | 1. Physiotherapists should follow clinical guidelines 2. Develop a standard practice guideline for Nigerian PTs |
|  | 1. Process modification | 1. Reschedule patients if the load is too much 2. The personnel should be available to carry out SC 3. Assessment and treatment on different days 4. Patients should visit smaller clinics where the clinicians have time 5. SBT can be filled at home 6. SBT can be sent over the internet 7. Short form with all questionnaires for patient assessment 8. Apportion sufficient time for each patient 9. Combine education with treatment 10. Blending TBS and orthodox practice |
|  | 1. Financial solutions | 1. If patients can get health insurance it can help 2. Institutions should train physiotherapists 3. Upgrade hospital with funding 4. Funds to print questionnaire |
|  | 1. Knowledge mobilisation | 1. Carry out research and present papers about SC 2. Training workshops can help to propagate SC in Nigeria |
|  | 1. Management solutions | 1. Awareness by hospital policy makers eases use of SC 2. Provide the right working environment 3. Supervision of therapists by hospital management |
|  | 1. Specialisations for physiotherapists | 1. Have more physiotherapist specialised 2. Implement through speciality groups |
|  | 1. Hierarchal implementation | 1. Implement through senior PTs 2. Physiotherapist supervision by senior colleagues |
|  | 1. Approach modification | 1. Making SC it attractive to use 2. Modify the approach to include activities patients enjoy |
|  | 1. Tool modification | 1. Language translations of SBT will make it easy for patients 2. Create a short version of the SBT 3. A special questionnaire for physical limitation |
|  | 1. Readjusting patient expectation | 1. Clinicians need to take time to understand patients 2. Going beyond the surface to help patients 3. Modify patients’ expectations using education 4. Make-shift treatment for patient psychology |
|  | 1. Re-adjusting patients’ attitude | 1. Patients have to be convinced 2. Co-operation with clinicians |
|  | 1. Educating patients | 1. Educating the non-patient population 2. Ensure that patients are educated 3. Patients need to educate themselves about their condition 4. Regulate information source |
|  | 1. Physiotherapist training | 1. Physiotherapists lack the necessary training on SC 2. Physiotherapists lack of knowledge about psychosocial intervention 3. Train physiotherapists together 4. Training is needed for physiotherapists to use psychosocial approach Include psychosocial training in undergraduate physiotherapy curriculum |
|  | 1. Communication with patients | 1. In SC the manner of presentation to patients is very important 2. Physiotherapists should communicate with the patient 3. Physios can apply this approach by being more attentive to yellow flags |
| Enablers | 1. Use of evidence-based practice | 1. Previous treatment done using physical assessment before treatment 2. Previous treatment using SC 3. Previous treatment using exercise therapy 4. Previous treatment using manual therapy 5. Previous treatment using patient education 6. Previous treatment using stratified care modifications |
|  | 1. Familiarity with approach | 1. Physiotherapists have background knowledge about psychosocial conditions 2. Physiotherapists in orthopaedic speciality might be familiar with a similar approach |
|  | 1. Confidence in the clinician | 1. Patients prefer to listen to physicians 2. Trust in the physiotherapist |
|  | 1. Patient willingness to accept change | 1. Patients open-mindedness 2. Slight improvement with treatment |
|  | 1. Self-care | 1. Self-management 2. Self-diagnosis by patient |
|  | 1. Experiences | 1. Lessons from trado-medical care 2. Role of religion |
| Outcome | 1. Practice benefits | 1. Reduces overtreatment of patients 2. Saves cost |
|  | 1. Knowledge benefits | 1. Opportunity for PTs to get knowledge 2. Physiotherapist will learn to use SC with experience 3. Understanding the approach |

TBS: Traditional bone setters; PIP: Psychologically informed physiotherapy; LBP: Low back Pain; SC: Stratified care; SBT: STarT-Back Tool; PT: Physiotherapist
