# Supplementary material for: Developing tailored intervention strategies for implementation of stratified care to low back pain with physiotherapists in Nigeria: a Delphi study
Source: BMC Health Serv Res. 2023 Feb 9;23:134. doi: 10.1186/s12913-023-09123-1 (PMC9909884; doi:10.1186/s12913-023-09123-1)
Supplement: Supplementary file 2 — Additional file 2. Individual Item Responses in Round 1 and Round 2. [file 12913_2023_9123_MOESM2_ESM.docx]

**Additional file 2: Individual Item Responses in Round 1 and Round 2**

| Statements: To implement Stratified Care, | Rounds | Strongly  Disagree (%) | | Disagree (%) | | Neutral (%) | Agree (%) | | Strongly Agree (%) | No Opinion (%) |  |
| --- | --- | --- | --- | --- | --- | --- | --- | --- | --- | --- | --- |
| *Group A: This group of statements deals with strategies to best modify stratified care management to fit the Nigerian context.* | | | | | | | | | | |  |
| 1. Start with senior PTs and let them supervise their junior colleagues. | Round 1 | | 0 | | 7 | 3 | | 38 | 50 | 0 | |
| 2. Hold quality improvement meetings to review successes and adherence. | Round 1 | | 0 | | 1 | 0 | | 25 | 71 | 0 | |
| 3. Educate the patients while treating them to save time | Round 1 | | 0 | | 2 | 4 | | 39 | 55 | 0 | |
| 4. Schedule assessment and treatment on two separate days. | Round 1 | | 1 | | 12 | 11 | | 38 | 38 | 0 | |
| **5. Translate the SBT into Nigerian pidgin. ^a^** | **Round 1** | | **2** | | **7** | **19** | | **33** | **39** | **0** | |
|  | **Round 2** | | **2** | | **15** | **12** | | **32** | **39** | **0** | |
| 6. Government should set up an affordable health insurance system | Round 1 | | 0 | | 3 | 6 | | 36 | 54 | 0 | |
| 7. Increase salary increments for PTs who train and practice PIP. | Round 1 | | 2 | | 5 | 9 | | 34 | 50 | 0 | |
| 8. Seek co-operation of the hospital administration. | Round 1 | | 2 | | 8 | 11 | | 39 | 40 | 0 | |
| **9. Implementation efforts should begin with government hospitals. ^a^** | **Round 1** | | **5** | | **16** | **18** | | **32** | **29** | **0** | |
|  | **Round 2** | | **5** | | **13** | **18** | | **34** | **30** | **0** | |
| 10. Patients should visit clinics with time and training on PIP | Round 1 | | 0 | | 7 | 6 | | 42 | 45 | 0 | |
| *Group B: This group of statements is concerned with participants views on how training and education can best be done to help the implementation of stratified care in Nigeria.* | | | | | | | | | | | |
| 11. Include training on PIP for undergraduate PTs | Round 1 | | 1 | | 2 | 1 | | 28 | 68 | 0 | |
| 12. Train PTs on pain-relieving medications | Round 1 | | 0 | | 7 | 13 | | 31 | 46 | 0 | |
| 13. Adopt standardised LBP treatment guideline in Nigeria. | Round 1 | | 1 | | 1 | 5 | | 28 | 65 | 0 | |
| 14. Group monitoring of colleagues is needed. | Round 1 | | 2 | | 7 | 9 | | 42 | 40 | 0 | |
| **15. TBS should share knowledge and ideas with PTs. ^a^** | **Round 1** | | **27** | | **20** | **18** | | **19** | **16** | **0** | |
|  | **Round 2** | | **35** | | **18** | **11** | | **18** | **18** | **0** | |
| 16. Training workshops on PIP for licenced physiotherapists | Round 1 | | 2 | | 2 | 2 | | 24 | 70 | 0 | |
| 17. PT speciality groups should take the responsibility of advocacy | Round 1 | | 0 | | 5 | 9 | | 43 | 42 | 0 | |
| 18. Inform other health professionals about SC | Round 1 | | 0 | | 3 | 5 | | 40 | 51 | 0 | |
| 19. Modify patients’ expectations by educating them on expected outcomes | Round 1 | | 0 | | 1 |  | | 28 | 68 | 0 | |
| 20. Use public media sources to dispel false information on LBP | Round 1 | | 2 | | 3 | 2 | | 34 | 59 | 0 | |
| 21. Encourage patients to learn about their own condition | Round 1 | | 0 | | 0 | 6 | | 27 | 64 | 0 | |
| 22. Monitor patients’ self-care routine | Round 1 | | 0 | | 2 | 5 | | 35 | 56 | 0 | |
| *Group C: This group of statements deal with ideas on conditions necessary to enhance the implementation of stratified care in Nigeria.* | | | | | | | | | | | |
| 23. PTs self-examination to remove wrong LBP beliefs and attitudes | Round 1 | | 0 | | 0 | 8 | | 41 | 49 | 0 | |
| 24. Use research results to convince PTs colleagues on SC | Round 1 | | 0 | | 0 | 4 | | 38 | 55 | 0 | |
| 25. Allocate sufficient time for PT-patient communication | Round 1 | | 0 | | 0 | 5 | | 40 | 55 | 0 | |
| **26. Patients should get a second opinion on the clinician’s advice. ^a^** | **Round 1** | | **1** | | **8** | **18** | | **44** | **29** | **0** | |
|  | **Round 2** | | **2** | | **12** | **14** | | **47** | **25** | **0** | |
| 27. Print STarT-Back Questionnaires for routine use in waiting rooms | Round 1 | | 0 | | 2 | 5 | | 36 | 56 | 0 | |
| **28. Physiotherapy consultations should be carried out online. ^a^** | **Round 1** | | **1** | | **12** | **17** | | **38** | **32** | **0** | |
|  | **Round 2** | | **2** | | **13** | **19** | | **35** | **31** | **0** | |
| 29. Provide an electronic version of the STB and fill online. | Round 1 | | 0 | | 2 | 8 | | 44 | 45 | 0 | |
| 30. Patients should fill the SBT at home. | Round 1 | | 1 | | 8 | 11 | | 38 | 42 | 0 | |

^a^ Statements not reaching consensus in Round 1 and progressing to Round 2.

TBS: Traditional bone setters; PIP: Psychologically informed physiotherapy; LBP: Low back Pain; SC: Stratified care; SBT: STarT-Back Tool; PT: Physiotherapist
