# Supplementary material for: Developing tailored intervention strategies for implementation of stratified care to low back pain with physiotherapists in Nigeria: a Delphi study
Source: BMC Health Serv Res. 2023 Feb 9;23:134. doi: 10.1186/s12913-023-09123-1 (PMC9909884; doi:10.1186/s12913-023-09123-1)
Supplement: Supplementary file 3 — Additional file 3. Qualitative data for Rounds 1 and 2. Common themes and indicative quotes. [file 12913_2023_9123_MOESM3_ESM.docx]

**Additional file 3: Qualitative data for Rounds 1 and 2. Common themes and indicative quotes.**

| **Category** | **Sub-category** | ***Quotes** |
| --- | --- | --- |
| **Round 1** | | |
| A) Strategies to best modify stratified care management to fit the Nigerian context. | Hierarchal implementation | - Implementation should be tested by a group of PTs on a group of patients first (F/BSc/<5/SpecialHosp). - Pilot the study and think of encouraging in-buy by giving instructions that implement incentives (M/PhD/20-25/SpecialHosp). - Collaboration with NDP and HODs very important (M/PhD/20-25/SpecialHosp) |
|  | Approach modification | - PT should adopt the use of Telemedicine in treating their patients (M/MSc/<5/HomePhys). - PT consultation should be carried out online sometimes if the patient is far away (F/MSc/25-30/HomePhys). |
|  | Tool modification | - Online administration of the SB tool (F/BSc/<5/TeachHosp). - Translated in different core ethnic languages, if possible (F/MSc/5-10/GenHosp). - A special questionnaire should be developed for the benefit of patients with visual and hearing impairments (M/BSc/5-10/HomPhys). |
| B) Views on how training and education can best be done to help the implementation of stratified care in Nigeria. | PT training | - Making SC a course for the undergraduate students (F/BSc/<5/GenHosp). - PT should be given equal opportunity to enhance their knowledge, skills and practice by engaging in MSc and PhD programs. (M/PhD/5-10/TrainInst). - PT should be trained on SC approach and increase sensitization (M/MSc/5-10/TeachHosp). - Adequate training of PT in pain care medication. Sensitization of the patient about this matching treatment plan (F/BSc/<5/HomePhysio). - SC should be included in undergraduate and postgraduate student training (M/PhD/5-10/TrainInst). |
|  | Educating patients | - By properly educating the patients and other professionals on the implementation of SC (M/MSc/<5/SpecialHosp). - Organisation of workshops and or seminars for PTs to educate them on the approach and benefits (M/BSc/<5/HomePhys). - Use of social media platforms (F/PhD/20-25/TrainInst). |
|  | Patient Communication | - Adequate follow up of patients in the course of the treatment (M/BSc/<5/HomePhys). - Involve patients with low back pain on their condition and treatment plan and should be carried along (F/BSc/5-10/SpecialHosp). |
|  | PT practice standardisation/regulation | - The licensing body should ensure more PTs are employed. This would improve Therapist to patients ratio significantly (M/MSc/5-10/TeachHosp) - To collaborate with the MRTB governing council to effect its implementation (M/BSc/10-15/SpecialHosp). |
|  | Re-adjusting patients’ expectations | - Cordial relationship with PT and their patient in order for them to open up (M/Bsc/5-10/SpecialHosp) - There should be shared decision making between the PT and the patient (M/BSc/<5/TeachHosp). |
| C) Ideas on conditions necessary to enhance the implementation of stratified care in Nigeria. | PT attitude re-adjustment | - I believe strongly that it begins with the PT. If he knows why it's important, he will use it (F/15-20/BSc/CommPhys) |
|  | Treatment tradition | - PTs should adopt the use of outcome measures while treating their patients (F/BSc/5-10/TeachHosp). - Extended scope practice should be encouraged among all specialist managing back pain especially the chronic low back (M/MSc/10-15/SpecialHosp). - PTs should have diagnostic authority such as basic laboratory and imaging tests (M/<5/MSc/Specialhosp). |
|  | Process modification | - Implement SC in rehab centre and home (M/BSc/<5/GenHosp). - PT should allocate more time in the assessment and treatment of patients with low back pain (M/Msc/<5/SpecialHosp). |
|  | Management solutions | - More PTs should be employed where this is being practised (M/PhD/5-10/HomePhys). - Patients load per PT should not be high (M/BSc/5-10/SpecialHosp). - Increase in PT workforce in our hospitals (M/BSc/5-10/TeachHosp) - Government policies and sensitization down to the grassroots (M/Bsc/5-20/CommPhys,HomePhys). |
|  | Interprofessional collaborations | - PTs to communicate with orthopaedic and musculoskeletal surgeons to understand the effect of SC on patient recovery especially on first contact (M/Msc/10-15/SpeciHosp) |
|  | Financial solutions | - Co-operation of HMOs and other health insurance companies should be sought to increase coverage and ensure provision of optimal health care to patients (M/Bsc/<5/TeachHosp). |
| **Round 2** | | |
| Familiarity with approach | No changes in opinions | - My answers did not change because I am well familiar with SB tool (M/PhD/5-10/TrainInst). |
|  |  | - I had no change in my choice (M/BSc/<5/GenHosp). - There are true representations of my options. These are the factors favourable for implementation (M/Bsc/10-15/GenHosp). |

* Format: Quote (Gender/Qualification/Years of experience/Work setting),

SC: Stratified care, SB: STarT-Back, PT: Physiotherapist, TrainInst: Training Institute, SpecialHosp: Specialist Hospital, TeachHosp: Teaching Hospital, CommPhys: Community Physiotherapy, HomePhys: Home Physiotherapy, GenHosp: General Hospital, SportCentr: Sports Center, PriHC: Primary Health care.
